# Prevalence of depression, syndemic factors and their impact on viral suppression among female sex workers living with HIV in eThekwini, South Africa

**DOI:** 10.1186/s12905-023-02392-2

**Published:** 2023-05-05

**Authors:** Anvita Bhardwaj, Carly A. Comins, Vijay Guddera, Mfezi Mcingana, Katherine Young, Rene Phetlhu, Ntambue Mulumba, Sharmistha Mishra, Harry Hausler, Stefan Baral, Sheree Schwartz

**Affiliations:** 1 Department of Mental Health, Johns Hopkins Bloomberg School of Public Health, Johns Hopkins Bloomberg School of Public Health, 624 N. Broadway, Baltimore, MD 21205 USA; 2 Department of Epidemiology, Johns Hopkins Bloomberg School of Public Health, Baltimore, MD 21205 USA; 3 TB HIV Care, Cape Town, South Africa; 4 University of Western Cape, Cape Town, South Africa; 5 Department of Medicine, University of Toronto, Toronto, ON USA; 6 MAP Centre for Urban Health Solutions, St. Michael’s Hospital, Unity Health Toronto, Toronto, ON USA; 7 Institute of Medical Science and Institute of Health Policy, Management and Evaluation, University of Toronto, Toronto, ON USA

**Keywords:** Female sex workers, Depression, South Africa, Syndemics

## Abstract

**Introduction:**

Over half of female sex workers (FSW) in South Africa are living with HIV and clinical depression has been frequently documented among FSW. Data characterizing structural determinants of depression and the role of syndemic theory, synergistically interacting disease states, on viral suppression among FSW in South Africa are limited.

**Methods:**

Between July 2018-March 2020, non-pregnant, cisgender women (≥ 18 years), reporting sex work as their primary income source, and diagnosed with HIV for ≥ 6 months were enrolled into the Siyaphambili trial in eThekwini, South Africa. Using baseline data, robust Poisson regression models were used to assess correlates of depression and associations between depression and syndemic factors on viral suppression.

**Results:**

Of 1,384 participants, 459 (33%) screened positive for depression, defined as a score of ≥ 10 on the PHQ-9. Physical and sexual violence, drug use, alcohol use, anticipated stigma and internalized stigma were univariately associated with depression (all *p*’s < 0.05) and included the multivariate model. In the multivariate regression, prevalence of depression was higher among participants experiencing sexual violence (PR = 1.47 95% CI:1.24,1.73), physical violence 5 times or more in < 6 months (PR = 1.38 95% CI:1.07, 1.80), using illicit drugs in the last month (PR = 1.23 95%:CI 1.04, 1.48), and reporting higher levels of internalized stigma (PR = 1.11, 95% CI:1.04,1.18). Depression in the absence of the Substance Abuse, Violence and AIDS SAVA syndemic factors was associated with increased prevalence of unsuppressed viral load (aPR 1.24; 95% CI:1.08,1.43), and the SAVA substance use and violence syndemic was associated with an increase in unsuppressed viral load among non-depressed FSW (aPR 1.13; 95% CI:1.01, 1.26). Compared to those experiencing neither factors, those jointly experiencing depression and the SAVA syndemics were at increased risk for unsuppressed viral load (aPR 1.15; 95% CI:1.02,1.28).

**Conclusion:**

Substance use, violence, and stigma were all associated with depression. Depression and syndemic factors (substance use + violence) were related to unsuppressed viral load; we did not observe higher unsuppressed viral load amongst those experiencing both depression and syndemic factors. Our findings point to the need to understand the unmet mental health needs of FSW living with HIV.

**Trial registration:**

Clinical Trial Number: NCT03500172.

**Supplementary Information:**

The online version contains supplementary material available at 10.1186/s12905-023-02392-2.

## Background

Female sex workers (FSW) are one of the key populations disproportionately affected by HIV, based on occupational and structural vulnerabilities. In low-and middle-income countries (LMICs), FSW face a 13.5 times greater odds of living with HIV when compared to all women of reproductive age [1]. Most data focus on FSW’s increased vulnerability to HIV and sexually transmitted infections; there are fewer data from LMICs assessing FSW vulnerability to psychosocial distress and their impacts on health outcomes [2–5].

FSW face increased levels of violence, childhood abuse, substance use, stigma, and social discrimination—all of which have been shown to be pathways to mental illness among FSW [1, 6, 7]. In LMIC settings, a recent systematic review found the pooled prevalence of psychological distress among FSW to be 40.8% [8]. In sub-Saharan Africa, studies show high levels of substance use among FSW and associations between substance use and poor physical health and cognitive outcomes [9–11]. Globally, FSW are specifically more vulnerable to depression than the general population due to these numerous risk factors [12–16]. It is estimated that the pooled prevalence of depression among FSW in LMICs is 41.8% [8].

Among varying groups living with HIV, depression has been shown to be a barrier to uptake and adherence to antiretroviral therapy (ART) [17–21]. Non-adherence to ART results in an individual being non-virally suppressed and, thus, results in poorer individual health outcomes including morbidity, potential for resistance to first line ART drugs and eventually mortality. At a population-level, persistent viremia may increase transmission [22]. Among the estimated 60% of FSW in South Africa living with HIV, only 39% are estimated to be on ART and viral suppression rates are poor though available data are limited [1, 23]. Resistance of ART regimens are a major concerns in LMICs especially due to limited number of ART regimens [24].

Due to high incidence of HIV in the population, along with high rates of substance misuse and violence led to theorization that FSW experience a syndemic, specifically the Substance Abuse, Violence and AIDS (SAVA) syndemic [25–27]. Syndemics are “the aggregation of two or more diseases or other health conditions in a population in which there are some level of deleterious biological or behavior interface that exacerbates the negative health effects of any or all the diseases involved” [28]. The theory focuses on looking at disease-disease and social condition-disease interactions [28]. Syndemics have played a large part of the HIV epidemic, with the first models outlined by Merril Singer focusing on gang violence, substance use and AIDS in the US [27, 29, 30]. The SAVA syndemic model has been the most utilized model to describe comorbidities among FSW in South Africa [31–33]. These studies have reinforced the evidence that co-occurring psychosocial conditions amongst FSW is associated with poorer HIV outcomes and increased risk of violence [31, 34]. Yet often the SAVA model is not teased apart to show what structural factors build up to make particular sections of the syndemic model components. Depression, in particular, may be modified by the SAVA syndemic factors of substance use and violence on HIV treatment outcomes. The effects of the SAVA syndemic on HIV viral suppression has only been tested among a few populations such as women of color, incarcerated individuals in the United States and sex workers in Kenya [35–37]. We hope to move the field of syndemics forward by looking at how depression might affect the SAVA syndemic amongst HIV positive FSW. As a percussor step to looking at the syndemic relationship, the stress-diathesis model was utilized to understand the factors that are associated with depression [38]. Previous work with this model in the field of HIV has shown that increased social support helps reduce the feeling of hopelessness amongst HIV positive mothers [39]. Using this model is helpful for us to understand the factors impacting depression in our population.

There is a growing awareness that interventions addressing the needs of FSW may need to address mental health concerns alongside structural determinants to improve health outcomes, including HIV prevention and treatment outcomes. This paper aims first to assess structural determinants of depression to inform the development of contextually appropriate mental health and HIV interventions, and secondly to assess the impact of depression and syndemics of depression, substance use and violence on viral suppression.

## Methods

### Study setting and population

Baseline data from the ongoing *Siyaphambili* trial in eThekwini (Durban), South Africa were analyzed. eThekwini is a metropolitan city in the Kwa-Zulu Natal Province in South Africa, the province with the highest HIV prevalence among women and girls in the world [40]. Study methods have been published in detail [41]. Briefly, non-pregnant, cisgender women (≥ 18 years), reporting sex work as their primary source of income and diagnosed with HIV for ≥ 6 months were eligible for enrollment into the 18-month adaptive intervention study. Participants were enrolled from July 2018 to March 2020. The date of the first registration was 17/04/2018 NCT03500172. At enrollment, those who were virally suppressed (viral load < 50copies/mL) continued on the standard of care, while non-virally suppressed participants were initially randomized into two intervention arms: (1) a decentralized treatment program; (2) peer-led individualized case management. Women in the study who completed the enrollment and baseline surveys were included in the analysis.

### Study procedures

Study implementation was conducted in tandem with an established program serving the HIV needs of FSW. Recruitment was led by the peer case managers on the research team at known sex work venues. The peer case managers on the research team had lived experience and were living with HIV. Peer case managers administered eligibility screening, the written informed consent process and enrollment. Blood draws (15 mL) were administered by a nurse and sent to the National Health Laboratory System for viral load assessment. Baseline surveys were conducted in isiZulu or English by trained interviewers in a private location at the TB HIV Care drop-in center for FSW or within the mobile van/private spaces at FSW venues and lasted on average 1-h and participants were reimbursed ZAR 100 (USD 7.50).

### Conceptual framework and measures

**Fig. 1:**
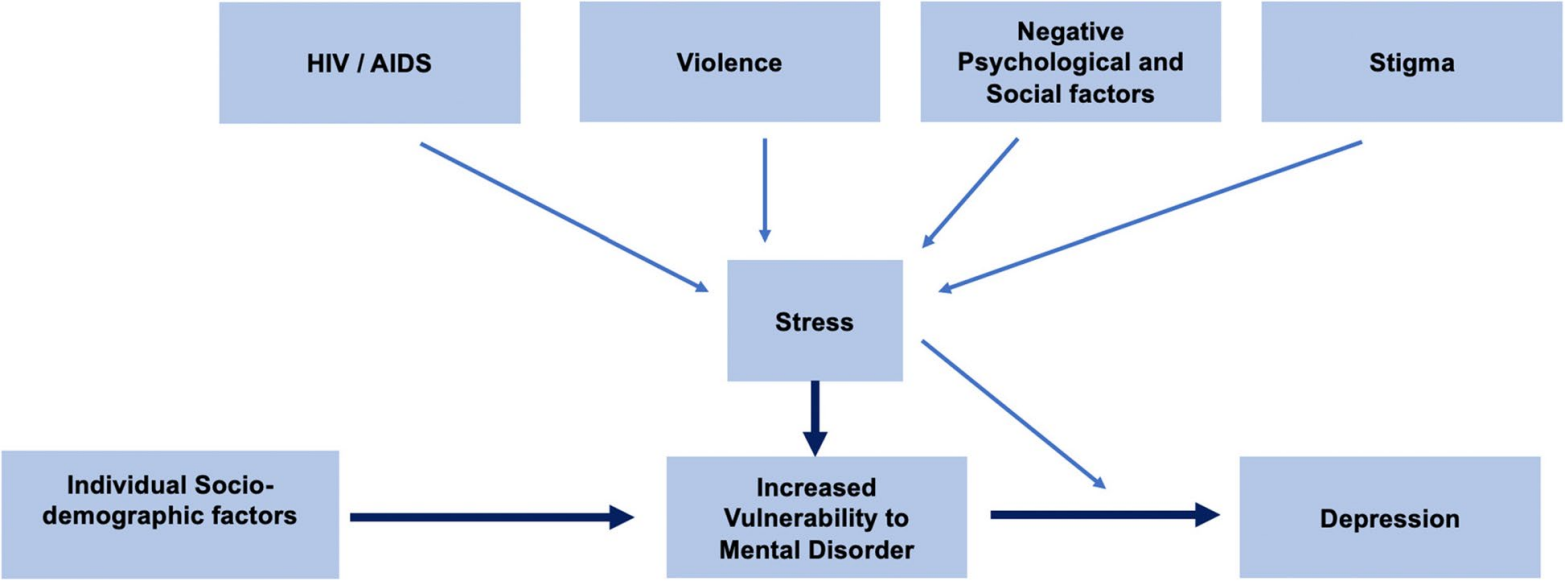
Adaptation of the Stress-Diathesis model from Kinyanda, E., Hoskins, S., Nakku, J., Nawaz, S., & Patel, V. (2011)^**^As all participants are HIV positive, the impact of HIV is measured by years since HIV diagnosis

An adaptation of the stress-diathesis conceptual model (Fig. 1) was used to guide analysis [42]. The premise of the conceptual framework is that specific factors (e.g. HIV diagnosis, experiences of violence and stigma) lead to stress, which in turn leads to increased vulnerability to mental disorders, such as depression. Although stress was not measured in this study, upstream factors leading to stress were collected. A limitation to the framework is that we do not take structural factors into consideration.

Available baseline measurements included: (1) Individual socio-demographic factors: age, highest educational level attained, relationship status, and income. Age was measured continuously. Education level was broken down into no formal education, those who completed some primary or secondary school, and those who completed secondary schooling or higher education. Relationship status was categorized as living with a steady partner, having a non-cohabitating steady partner, and single. Income was split into tertiles (≤ 1,500 Rand, 1500–3,000 Rand, and > 3,000 Rand per month). (2) HIV/AIDS: the effect of HIV was assessed through years since HIV diagnosis and split into three categories. (3) Violence: both sexual and physical violence were assessed. Sexual violence was measured as history of lifetime rape (yes/no). Physical violence was separated into three categories: No experience of violence in the last 6 months, experienced physical violence < 5 times in the last 6 months, and experienced physical violence ≥ 5 times in the last 6 months. (4) Items that fit the category of negative psychological and social factors were included based on previous use of the framework and included substance use, homelessness, social support, and years involved in sex work [42]. Substance use was assessed through self-reported drug and alcohol use. If any type of illicit drugs were used within the last month that was considered positive for drug use. Alcohol use was measured using the 3-question version of the AUDIT-C; with a score of 3 + being positive for hazardous alcohol use [43]. Social support was assessed using the Medical Outcomes Study Social Support Scale and modeled based on tertiles (low, medium and high); simple imputation using the average item score for an individual was used for women missing 5 or fewer social support items [44]. Homelessness was a binary variable (living on the street, in an abandoned house, or in a shelter). The number of years in sex work was modeled as a binary variable, divided at the median of 5 years. (5) Stigma was divided into anticipated (6 items), perceived (9 items), internalized (3 items) and enacted (13 items) stigma (see Table A.1) [45]. Continuous integer scores were calculated for each type of stigma [46]. If a participant responded yes to a question, that was scored as one point [46]. If they responded no or did not know that was scored as a zero [46]. The questions pertained to stigma towards living with HIV and being a FSW (see table A1). Finally, the outcome of depression was measured using the PHQ-9 included with a cut off score of 10 or higher identifying someone to be screened positive for exhibiting symptoms of depression and who would benefit from clinical care [47]. We utilized locally weighted smoothing scatterplots and measures of the median to come up with the appropriate cutoffs. We used multiple imputation to address missingness for variables other than social support which used the method described above.

### Data analysis

Prevalence of baseline depression in the cohort was described and evaluated across sociodemographic factors and covariates under the guiding stress diathesis framework. Next regression models were run to separately assess depression and viral suppression outcomes. Women who were missing all or the majority of the baseline questionnaire (*n* = 19) were not included. Locally weighted polynomial regressions were used to assess functional forms of the relationships between continuous variables and the outcome. Prevalence ratios (PR) and 95% confidence intervals (95% CI) were calculated as the primary measures of association using modified robust Poisson regression as the prevalence of the outcomes was greater than 10% and log-binomial models did not converge [48]. Firstly, univariate models assessed associations between potential upstream factors (based on the modified stress-diathesis model) and the dependent variable depression. If a factor was showed evidence of a statistical trend at *p* < 0.1 in the univariate models, the variable was included in the multivariate, age-adjusted model. Based on previous work from the SAVA syndemic model, a priori we hypothesized that violence and substance use may work together to modify the relationship between depression and viral suppression. We thus assessed the relationships independently and jointly between depression and the SAVA syndemic factors (modeled as those experiencing substance use and violence) adjusting for age, homelessness partnership status, and anticipated stigma based on correlates of depression as well as the viral suppression outcomes within the dataset as well as literature review. Independent and joint PRs were reported from the adjusted models. In both multivariable outcome models (depression and unsuppressed viral load), we assessed collinearity across variables in the model by estimating the variance inflation factors and used multiple imputation using chained equations to address missingness for variables [49]. All statistical analysis was done using StataSE 15.0 (College Station, Texas, USA) [50].

### Ethical approvals

All study procedures were approved by the University of the Western Cape Biomedical Research Ethics Committee, the Johns Hopkins School of Public Health Institutional Review Board, the KwaZulu-Natal Department of Health and the eThekwini municipality. All experiments were performed in accordance with the Declaration of Helsinki.

## Results

### Participant characteristics and prevalence of depression

Women enrolled in Siyaphambili (*N* = 1,384) were a median age of 31 years [IQR 27–37]. About half of the women were single at baseline (*n* = 701, 50.6%). Women’s total income was a median of 2,000 South African Rand per month [IQR 1,000–3,400] (equivalent of USD 150). Overall, 33.2% (*n* = 459) of enrolled women screened positive for depression (Table 1). Similar sociodemographic trends were observed between those with and without depression.

**Table 1:** Sociodemographic characteristics of female sex workers living with HIV in Durban, South Africa (*n* = 1,384)

Characteristics	Positive screen for depression (*n* = 459)	Negative screen for depression (*n* = 925)	*p*-value
	**Median [IQR]**	**Median [IQR]**	
Age*	31 (27,37)	31 (27,36)	0.627
	***n* (%)**	***n* (%)**	
**Education Level**			0.447
No formal education	46 (10.0)	74 (8.0)	
Some primary or secondary education	325 (71.0)	673 (72.8)	
Completed secondary education and higher	88 (19.0)	178 (19.2)	
**Relationship Status**			0.152
Steady partner living together	62 (13.5)	148 (16.0)	
Steady partner not living together	148 (32.2)	325 (35.1)	
Single	249 (54.3)	452 (48.9)	
Work Venue Type^*^			0.226
Indoor (dwelling of some type)	326 (71.2)	686 (74.2)	
Outdoor (park, truck stop)	132 (28.8)	238 (25.8)	
Monthly Income (ZAR)**			0.399
Up to 1,500	184 (40.6)	340 (38.0)	
1500 to 3,000	142 (31.6)	315 (35.3)	
3,000 +	126 (27.8)	239 (26.7)	

Depression is defined as a PHQ score ≥ 10; p-values estimated using Wilcocon rank-sum for comparisons of medians and X^2^ statistics for comparisons of categorical variables. *2 participants were missing a response in age, type of work venue. **ZAR = South African Rand (~ 13.9 Rand/USD at the time of enrollment); 37 women were missing income

### Social and structural factors and correlates of depression

Overall, 46.9% (*n* = 649) of participants were diagnosed with HIV more than five years ago and the majority had been involved in sex work for more than five years (*n* = 799, 57.7%). Nearly one-third of women (*n* = 412, 29.9%) reported homelessness at enrollment. As depicted in Table 2, physical violence, sexual violence, severe risk in the AUDIT-C, drug use, internalized stigma and anticipated stigma were all associated with depression in the univariate models at the *p* < 0.1 level and thus were included in the multivariate model.

**Table 2:** Prevalence of social and structural correlates and associations with depression among female sex workers living with HIV in Durban, South Africa (*n* = 1,384)

	**Overall proportion with exposure (*n* = 1,384)**	**Crude PR for Depression (95% Cl)**	***p*-value**
	*n* (%)		
**HIV / AIDS**			
Years since HIV diagnosis **			
1 year or less	204 (14.8)	REF	
1 to 5 years	531 (38.4)	0.85 (0.68, 1.06)	0.15
Greater than 5 years	649 (46.8)	0.97 (0.78, 1.20)	0.77
**Violence**			
Physical Violence^#^			
Never or not in the last 6 months	638 (46.4)	REF	
1–5 times in the last 6 months	638 (46.4)	1.36 (1.16, 1.60)	< 0.01
More than 5 times in the last 6 months	97 (7.2)	1.84 (1.44, 2.33)	< 0.01
Ever experienced sexual violence (rape)	520 (37.6)	1.64 (1.41, 1.90)	< 0.01
**Negative Psychological and Social Factors**			
Homeless^##^	412 (29.9)	1.11 (0.95, 1.31)	0.18
Low Social Support vs High/medium social support	480 (34.7)	1.01 (0.86, 1.18)	0.92
Illicit drug use within the last month^+^	866 (63.6)	1.27 (1.08, 1.49)	< 0.01
AUDIT-C Risk Category^¥^			
Low Risk	399 (40.1)	REF	
Moderate to high risk	261 (26.3)	1.06 (0.86, 1.30)	0.59
Severe Risk	334 (33.6)	1.22 (1.02, 1.46)	0.03
Years involved in sex work			
5 years or less	585 (42.3)	REF	
More than 5 years	799 (57.7)	0.97 (0.83, 1.13)	0.73
**Median [IQR]**			
**Stigma**^‡^			
Anticipated Stigma	1 [0,1]	1.05 (1.00, 1.11)	0.05
Perceived Stigma	1 [0,1]	1.01 (0.96, 1.07)	0.70
Enacted Stigma	1 [0,1]	1.02 (0.96, 1.10)	0.43
Internalized Stigma	1 [0,2]	1.13 (1.04, 1.19)	0.01

*PR* = *Prevalence ratio; estimated from modified robust Poisson regression models;* Depression is defined as a PHQ score ≥ 10

^**^missing for *n* = 21; # missing for *n* = 11; ## missing for *n* = 2; + missing for *n* = 23; ¥ Missing full Audit-C for *n* = 390 participants due to randomly administered survey error; ‡Stigma was measured as a continuous score. Anticipated stigma was comprised of 6 items, perceived stigma 9 items, enacted stigma 13 items, and internalized stigma 3 items (Table A1)

In the age-adjusted, multivariate model (Table 3), prevalence of depression was associated with having experienced physical violence more than five times in the past 6 months (aPR = 1.38, 95% CI: 1.07, 1.80) as compared to experiencing no physical violence in the last 6 months. Having any lifetime experience of sexual violence (aPR = 1.47 95% CI: 1.07, 1.80) and using illicit drugs in the past month (aPR = 1.23, 95% CI: 1.04, 1.48) were also associated with depression. Higher levels of internalized stigma were also associated with depression (aPR = 1.11, 95% CI: 1.04, 1.18).

**Table 3:** Multivariable modified robust Poisson regression model of correlates for depression with HIV positive female sex workers in eThekwini, South Africa (*n* = 1384)

	**Adjusted Prevalence Ratios for Depression (95% Cl)**	***p*-value**
**Physical Violence**		
Never or not in the last 6 months	REF	
5 times or less in the last 6 months	1.16 (0.98, 1.38)	0.081
More than 5 times in the last 6 months	1.38 (1.07, 1.80)	0.01
**Ever experienced sexual violence**	1.47 (1.24, 1.73)	< 0.01
**AUDIT-C Risk Category**^**¥**^		
Low Risk	REF	
Moderate to high risk	1.10 (0.88, 1.37)	0.39
Severe Risk	1.20 (0.98, 1.46)	0.08
**Illicit drug use in the past month**	1.23 (1.04, 1.48)	0.02
**Internalized Stigma**^**#**^	1.11 (1.04, 1.18)	< 0.01
**Anticipated Stigma**^**#**^	1.00 (0.95, 1.06)	0.84

Depression is defined as a PHQ score ≥ 10

^*^*Age-adjusted models control for age using a quadratic term (age + *age*^*2*^*)*

^**¥**^the 3 question AUDIT-C scale was used

^*#*^Stigma was measured as a continuous score. Anticipated stigma had 6 questions, and internalized stigma had 3

When assessing the associations between depression and the joint syndemics term including substance use and violence on viral suppression, both depression and SAVA were independently significantly associated with a higher prevalence of viremia (Table 4). Among FSW not reporting substance use and violence syndemics, depression was associated with a 24% increase in prevalence of unsuppressed viral load (aPR 1.24; 95% CI: 1.08, 1.43), whereas the SAVA syndemic (substance use and violence) was associated with a 13% estimated increase in unsuppressed viral load among those who were not depressed (aPR 1.13; 95% CI: 1.01, 1.26). Among those jointly experiencing depression and the SAVA syndemics, unsuppressed viral load was 15% higher than those experiencing neither factor (aPR 1.15; 95% CI: 1.02, 1.28). This was done through interaction terms.There was a statistically significant antagonistic (negative) modification of effect between depression and SAVA such that the joint effects were less together than what was experienced independently (aPR: 0.81, 95% CI: 0.68, 0.97; *p* = 0.020).

**Table 4:** Adjusted Correlates between the SAVA syndemic factors and unsuppressed viral load among female sex workers living with HIV in eThekwini, South Africa (*n* = 1384)

	**Prevalence Ratios for unsuppressed viral load (95% CI)**	***p*-value**
Depression in the absence of substance use and violence	1.24 (1.08, 1.43)	< 0.01
SAVA syndemic (substance use and violence) in the absence of depression	1.13 (1.01, 1.26)	0.01
Depression and SAVA syndemic (substance use and violence)^#^	1.15 (1.02, 1.28)	0.02
No depression and absence of substance use and violence syndemic	REF	–

Modified robust Poisson regression models adjusted for age (as a cubic term), homelessness, relationship status, and anticipated stigma were used to estimate adjusted prevalence ratios (aPRs)

^*^not virally suppressed is defined as viral load > 50copies/mL; the model was adjusted for age, homelessness and relationship status. Depression is defined as a PHQ score ≥ 10

+ the SAVA syndemic term was a combination of (i) ever experiencing physical or sexual violence and (ii) being in the moderate, high or severe risk category on the AUDIT-C or using illicit drugs in the past month

^#^modeled as an interaction term

## Discussion

This analysis highlights the vulnerability to depression and upstream structural determinants of depression among HIV positive FSW in eThekwini, South Africa. Syndemic theory and the stress-diathesis framework were used as the foundation to understand these factors. Substance use, experiencing frequent physical violence, any sexual violence, and internalized stigma were all independently associated with a higher prevalence of depression. Depression was also associated with lower viral suppression, as was the joint syndemic of violence and substance use.

In the Siyaphambili cohort, 32.9% of the women screened positive for having depression, which is considerably higher than the reported rate of depression of 4.9% in the total adult population of South Africa [8, 51]. Past studies have shown similar values with a 2003 study showing 34.9% of people living with HIV screening positive for depression in South Africa [52]. Limited studies have looked specifically at depression among FSW in South Africa. The prevalence seen in our cohort is lower than that seen from other FSW in Kwa-Zulu Natal (80.6%) that also use the PHQ-9, yet the Poliah & Paruk^14^ study drew from FSW who were attending a support group and thus may represent a biased sample [53]. FSW in Soweto had a higher prevalence of depression than our cohort at 66.3%, but used a different scale to measure depression [54]. Importantly, our analysis highlights that experiences of physical and sexual violence, drug use, and internalized stigma are key structural factors associated with depression in the cohort. Findings from our study support those seen in Entebbe Uganda, where the stress-diathesis framework was also used and identified psychological and negative social factors as the main section of the framework associated with depression among people living with HIV [42, 55].

Exposure to frequent physical and any history of sexual violence were both strongly associated with an increased prevalence of depression. Increased vulnerability to violence and high levels of violence experienced among FSW in South Africa is well documented [54, 56, 57]. In a recent meta-analysis among FSW in LMICs, significant associations were seen between experiences of violence and depression [8]. In Kwa-Zulu Natal, high levels of violence towards FSW can be partially attributed to the cultural expression of masculinity and male domination in the region [58]. The association between ever experiencing sexual or physical violence on depression is well documented among FSW worldwide such as in the Caribbean, Mexico, the Gambia, South Africa and India [59–62]. Among women in the United States, it has been shown that more recent physical or sexual violence exposure led to more than a two-fold higher prevalence of depressive symptoms [63]. Most studies with FSW look at childhood violence and experiencing violence anytime in adulthood, rather than looking at more proximal adult exposures [16, 62, 64]. Our findings show a relationship between the extent of recent physical violence and depression, reinforcing the importance of screening for recency and frequency of physical violence in similar populations.

High rates of internalized stigma have been seen in similar HIV positive FSW populations, including in Zimbabwe, Tanzania and the Dominican Republic [65–67]. Though other studies have shown that FSW living with HIV reported higher internalized stigma than HIV negative FSW, within our cohort we were unable to identify these dynamics of intersectional stigma as all women were living with HIV [66]. Depression and internalized stigma have both been shown to result in poor ART adherence and treatment outcomes for FSW with HIV, thus pointing to the need for interventions to target both stigma and poor mental health [68, 69]. A study in Johannesburg modeled that implementing interventions to promote ART adherence for FSW could lead to a 2% absolute drop in the district’s incidence rate of new HIV infections [22].

Our models looking at the joint effect of the SAVA syndemic and depression on viral suppression illustrated that depression was independently associated with lower viral suppression. Viral suppression was also lower among those experiencing substance use and violence together in the absence of depression. The combination of substance use, violence, and depression jointly, was associated with greater viral suppression as compared to those experiencing neither depression nor the SAVA syndemic factors, however the joint association was lower than the independent effects. It is possible that among those experiencing both depression and SAVA, the effects were also correlated to homelessness and stigma and thus attenuated in a fully adjusted model. A longitudinal study in Kenya did not see any relationship between the SAVA syndemic and viral suppression [36]. Among women of color with HIV in the United States, high values on the SAVA score were associated with lower odds of viral suppression; yet a plateau effect was seen after experiencing three or more psychosocial problems [37]. Those who experience all components of the SAVA syndemic including depression may face a broader set of challenges that were not measured as a part of our model, though overall this group of individuals still had a significant increase in unsuppressed viral load compared to those who were not depressed or experiencing SAVA syndemic factors.

The key takeaway from this analysis is that there are factors and experiences that may help identify FSW in South Africa and similar settings who are at risk of developing or experiencing undiagnosed and untreated depression, which may also impact HIV treatment outcomes. These data may also inform the development of mental health prevention interventions, where the goal is to prevent poor mental health outcomes in a population. Our findings may support the identification of women at the highest need for an indicated prevention program, as they are possibly predisposed to being at higher risk of developing depression [31, 70, 71]. Given the relationship seen between depression and unsuppressed viral load, preventative interventions that reduce the prevalence of depression amongst FSW may also support reductions in onward HIV transmission [18, 20]. Along with prevention programs, our findings can support interventions that integrate mental health and HIV care through the identification of those most at risk of having those comorbidities. Community platforms and the use of case management programs delivered by non-mental health professionals could be tailored for at risk populations [72]. Pilot studies have shown that it is feasible to integrate mental health, interpersonal violence and HIV care together in Cape Town and this is currently being scaled up [31].

This analysis has limitations. Given the cross-sectional design of the study, temporality between depression and upstream risk factors cannot be assessed. Additionally, our entire population was living with HIV, and hence we were not able to make comparisons to those not living with HIV. We also had a large amount of missing data for the AUDIT-C scale. In regards to the stress-diathesis framework, a key limitation is that stress was not directly measured, however we were able to measure variables that have been observed to lead to stress in other settings. Other potentially important negative psychological influences, such as PTSD and childhood abuse, were not measured.

## Conclusion

The results suggest that risk factors for depression among HIV positive FSW in South Africa include experiences of frequent physical violence, sexual violence, drug use, and internalized stigma. Individuals reporting these factors, even in the absence of depressive symptoms, may be considered for mental health support. Given the association between viral suppression and depression as well as syndemic factors, broad screening and support are indicated. Integration of HIV and mental health interventions may take time. In the interim, HIV interventions facilitated by non-mental health providers, such as peer case managers, may be adapted to screen for these risk factors and proactively refer women to mental health care to support in the prevention and treatment of depression and strategies to address ongoing vulnerabilities such as substance misuse and violence or trauma. Given the multiple layers of marginalization, FSW are at a unique risk for sub-optimal health outcomes and interventions may need to tailor to the specific vulnerabilities among FSW to efficiently and effectively allocate resources to improve overall health and HIV outcomes.

## Supplementary Information

**Additional file 1.**

## Data Availability

The datasets used and analyzed during the current study are available from the corresponding author on reasonable request.
