# Correction to: Prevalence of depression, syndemic factors and their impact on viral suppression among female sex workers living with HIV in eThekwini, South Africa

**DOI:** 10.1186/s12905-023-02433-w

**Published:** 2023-05-19

**Authors:** Anvita Bhardwaj, Carly A. Comins, Vijay Guddera, Mfezi Mcingana, Katherine Young, Rene Phetlhu, Ntambue Mulumba, Sharmistha Mishra, Harry Hausler, Stefan Baral, Sheree Schwartz

**Affiliations:** 1 Department of Mental Health, Johns Hopkins Bloomberg School of Public Health, Johns Hopkins Bloomberg School of Public Health, 624 N. Broadway, Baltimore, MD 21205 USA; 2 Department of Epidemiology, Johns Hopkins Bloomberg School of Public Health, Baltimore, MD 21205 USA; 3 TB HIV Care, Cape Town, South Africa; 4 University of Western Cape, Cape Town, South Africa; 5 Department of Medicine, University of Toronto, Toronto, ON USA; 6 MAP Centre for Urban Health Solutions, St. Michael’s Hospital, Unity Health Toronto, Toronto, ON USA; 7 Institute of Medical Science and Institute of Health Policy, Management and Evaluation, University of Toronto, Toronto, ON USA

Following publication of the original article [1], the affiliations 3 and 4 has been updated has shown below:

^3^TB HIV Care, Cape Town, South Africa.

^4^University of Western Cape, Cape Town, South Africa.

The original article has been corrected.

