# Supplementary material for: Prevalence of depression, syndemic factors and their impact on viral suppression among female sex workers living with HIV in eThekwini, South Africa
Source: BMC Womens Health. 2023 May 5;23:232. doi: 10.1186/s12905-023-02392-2 (PMC10161481; doi:10.1186/s12905-023-02392-2)
Supplement: Supplementary file 1 — Additional file 1. [file 12905_2023_2392_MOESM1_ESM.docx]

**APPENDIX**

***Table A. I. Stigma Questions included in Analysis and organized by type of stigma***

| **Question** | **Type of Stigma** |
| --- | --- |
| Have you ever been afraid to seek healthcare because someone might learn that you are a sex worker? | Anticipated |
| Have you ever avoided seeking healthcare because you were afraid someone might learn that you are a sex worker? | Anticipated |
| Have you ever been afraid to be in public places because you are FSW? | Anticipated |
| Have you ever been afraid to seek healthcare because someone might learn that you are living with HIV? | Anticipated |
| Have you ever avoided seeking healthcare because you were afraid someone might learn that you are living with HIV? | Anticipated |
| Have you ever been afraid to be in public places because you are living with HIV? | Anticipated |
| Have you ever been denied health services or have someone keep you from receiving health services because you are a sex worker? | Enacted |
| Have you ever felt that you were verbally harassed because you’re FSW? | Enacted |
| Have you ever felt that you were blackmailed because you are living with HIV? | Enacted |
| Have you ever told a family member that you are a sex worker? | Enacted |
| Have you ever felt that a family member made a negative remark or gossiped about you because you are a sex worker? | Enacted |
| Have you ever avoided carrying condoms because you thought they might cause you problems from a uniformed officer? | Enacted |
| Have you ever seen a uniformed officer take or destroy condoms carried by you or another sex worker? | Enacted |
| Have you ever been arrested on charges related to prostitution? | Enacted |
| Have you ever felt that you were blackmailed because you are FSW? | Enacted |
| Have you ever told a family member that you are living with HIV? | Enacted |
| Have you told your husband/boyfriend/non paying partner ? | Enacted |
| Have you ever been denied health services or have someone keep you from receiving health services because you are living with HIV? | Enacted |
| Have you ever felt that you were verbally harassed because you are living with HIV? | Enacted |
| Selling sex is a satisfactory and acceptable way of life for me. | Internalized stigma |
| For the most part, I do not care if people know I sell sex. | Internalized stigma |
| Selling sex does not make me a lesser person. | Internalized stigma |
| Have you ever felt excluded from family activities because you are a sex worker? | Perceived |
| Have you ever felt rejected by your friends because you are a sex worker? | Perceived |
| Have you ever felt that you were not treated well in a healthcare center because you are a sex worker?  *For example: waiting longer, being isolated or misguided, not receiving the quality of care that you deserve, etc.* | Perceived |
| Have you ever felt that a healthcare worker made negative remarks or gossiped about you because you are a sex worker?  *Healthcare worker: Doctor, nurse, social worker, etc* | Perceived |
| Have you ever felt that a uniformed officer refused to protect you because you are a sex worker?  *Uniformed officer: Police, soldier, etc.* | Perceived |
| Have you ever felt a uniform officer harassed or intimidated you because you are a sex worker? | Perceived |
| Have you ever felt excluded from family activities because you are living with HIV? | Perceived |
| Have you ever felt that a family member made a negative remark or gossiped about you because you are living with HIV? | Perceived |
| Have you ever felt rejected by your friends because you are living with HIV? | Perceived |
| Have you ever felt that you were not treated well in a healthcare center because you are living with HIV?  *For example: waiting longer, being isolated or misguided, not receiving the quality of care that you deserve, etc.* | Perceived |
| Have you ever felt that a healthcare worker made negative remarks or gossiped about you because you are living with HIV?  *Healthcare worker: Doctor, nurse, social worker, etc.* | Perceived |
